# Beyond the burn: Studies on the physiological effects of flamethrowers during World War II

**DOI:** 10.1186/s40779-020-00237-9

**Published:** 2020-02-27

**Authors:** David W. Van Wyck

**Affiliations:** 4/3 SFG (A) Bldg Z-4157 South Post Rd, Fort Bragg, NC 28310 USA

**Keywords:** Flamethrower, Burns, Carbon monoxide, Asphyxiation, Hypoxia, Chemical warfare

## Abstract

Flamethrowers are widely considered one of warfare’s most controversial weapons and are capable of inflicting gruesome physical injuries and intense psychological trauma. Despite being the last of the major combatants in World War II (WWII) to develop them, the United States military quickly became the most frequent and adept operator of portable flamethrowers. This gave the U.S. military ample opportunity to observe the effects of flamethrowers on enemy soldiers. However, while most people in modern times would consider immolation by flamethrower to be an unnecessarily painful and inhumane way to inflict casualties, immolation was, at one point during World War II (WWII), referred to as “mercy killing” by the U.S. Chemical Warfare Service (CWS). This mischaracterization arose from a series of first-hand accounts describing what were believed to be quick, painless, and unmarred deaths, as well as from a poor and incomplete understanding of flamethrower lethality. As a result, indirect mechanisms such as hypoxia and carbon monoxide poisoning were generally absent from accounts of the flamethrower’s fatal effects. It was not until several years after flamethrowers were introduced to the frontlines that the CWS and National Defense Research Committee (NDRC) conducted a series of tests to better understand the physiological and toxicological effects of flamethrowers. This article examines how the initial absence of scientific data on the physiologic effects of flamethrowers led to an inaccurate understanding of their lethality, and bizarre claims that one of history’s most horrific instruments of war was considered one of the more “humane” weapons on the battlefield.

## Background

Despite the widespread development, manufacturing, and use of flamethrowers by the U.S. military in the Pacific theater during World War II (WWII), there was surprisingly little effort directed at determining details on the lethality, physiological and toxicological effects of these weapons. Although flamethrowers grew in popularity in the Pacific, where they were employed against enemy soldiers known to take up strongly fortified and entrenched positions, there seemed to be a lack of scientific interest in studying the weapon, with one *Chemical Warfare Bulletin* article from 1944 stating, “It was felt that so long as the flamethrower produced death to the enemy, the precise physiological reaction or how soon death supervened were immaterial [1].” However, with the increased use of the flamethrower in the Pacific theater, there came a growing interest in developing better tactics to maximize the potential of the weapon. This required, in part, a better understanding of how the flamethrower produced casualties. Moreover, increased utilization meant increased publicity, and the nature of a flame weapon caused controversy. Newspapers increasingly referred to flamethrowers as barbarous, inhumane weapons of horror, and the Japanese were described as being terrified of the weapon, with some accounts reporting Japanese soldiers taking their own lives when faced with an imminent flamethrower attack [2]. Contrasting portrayals were published by the U.S. military that included first-hand accounts from U.S. chemical soldiers and officers citing not only the effectiveness of the flamethrower on fortified enemy positions but also observations that the weapons seemingly produced instantaneous deaths, even in situations where there was little or no evidence of thermal injury on enemy corpses. Some went so far as to claim that flamethrowers were “mercy killers,” particularly when compared to bullets and high explosives [3]. All these considerations prompted the Chemical Warfare Service (CWS), the National Defense Research Committee (NDRC), the Medical Corps, and a host of other private companies and universities to conduct experiments on the toxicology and physiology of flamethrower attacks that better assessed the military utility and killing power of this terrifying weapon. These studies helped end the notion of flamethrowers as mercy weapons and provided physiologic and toxicologic data on burns, heat exposure thresholds, asphyxiation, inhalational injury from smoke and chemical irritants, and carbon monoxide and dioxide poisoning that remain useful in contemporary military medicine.

### Overview of portable flamethrowers

**Fig. 1:**
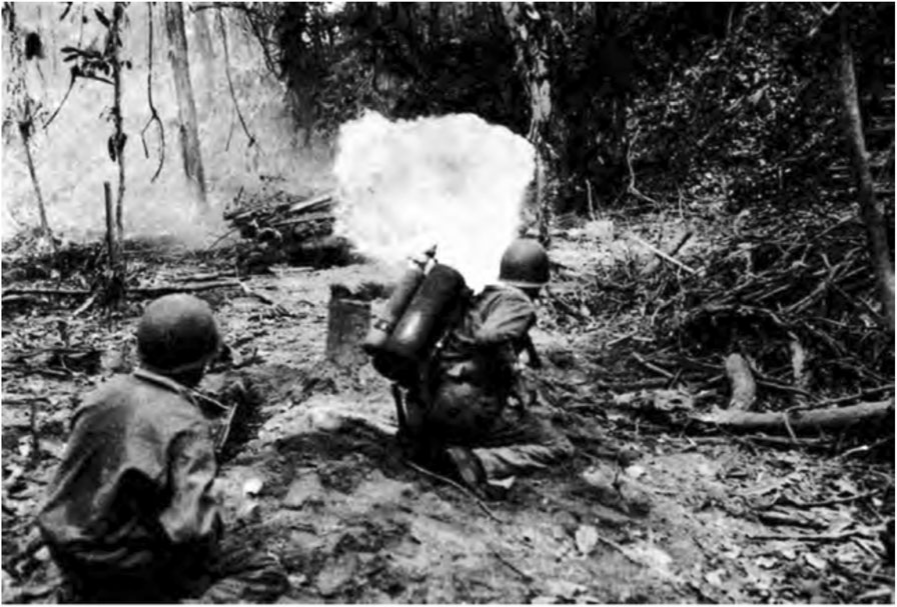
An M1A1 flamethrower used by U.S. Army soldiers to clear a Japanese Bunker on Bougainville Island in March of 1944. Photo from Brophy, Miles and Cochrane, The Chemical Warfare Service: From Laboratory to Field, Department of the Army, 1959

**Fig. 2:**
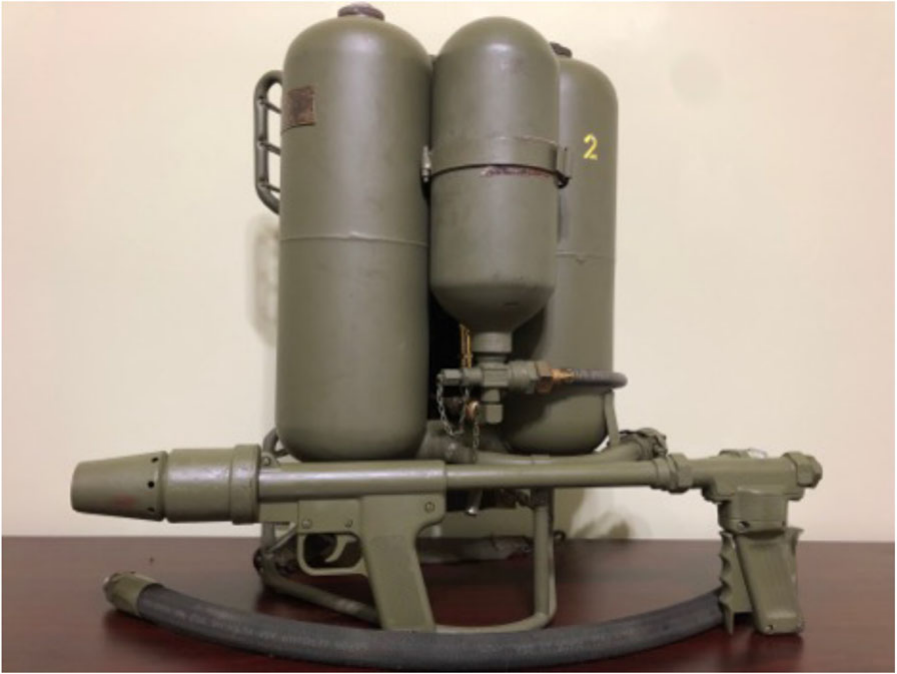
The M2–2 man-portable flamethrower utilized by the U.S. military in the Pacific theater during the last 2 years of WWII. The example is working unit from the author’s personal collection. The original hex shaped gas caps, rupture disks and vent tube have been replaced with the more reliable and safe vented fuel caps developed in 1953

While the use of fire in warfare has existed since approximately 424 BC when the Greeks created the first flamethrower, it was not until World War I (WWI) that flame warfare was adapted for use by soldiers in a portable fashion. The Germans developed the first man-portable flamethrowers and flame warfare tactics, and other nations soon followed suit. As the conflict progressed from static to mobile combat in the final year of the war, there were fewer opportunities to effectively use the flamethrower. Because the U.S. did not enter the war until 1918, the country gained little experience with flamethrowers and unsurprisingly failed to study or develop flamethrowers of its own during the interwar years. It was not until July of 1940, after the U.S. military became aware of the use of flamethrowers in Europe during the early part of WWII, that the Secretary of War ordered the CWS to develop a flamethrower. Interestingly, while the U.S. was the farthest behind of all major combatants in the development of flamethrowers and arguably produced the most onerous flamethrower of the war, the M1 portable flamethrower (Fig. 1), U.S. soldiers quickly became the most adept users of flamethrowers and relied heavily on their use in the Pacific theater. In a short period of time, the U.S. would eventually develop what is widely considered to be the best flamethrower of the war, the M2–2 flamethrower (Fig. 2). Yet despite the focus on developing an effective flamethrower and flamethrower tactics, the U.S. had a poor initial understanding of the weapon’s lethality [4]. According to opening statements made at the 1945 “Symposium on the Toxicological Aspects of Flame Throwers” by Major General William N. Porter, Chief of the U.S. CWS, “the failure of the nation to support a strong and active military scientific organization during the peace time years was a great handicap to us when the need for rapid preparation for this war became apparent [5].” After rushing the weapons into development and onto the battlefield with little to no scientific data, several first-hand observations began to appear in military reports that mischaracterized the weapon’s use and effects.

### The flamethrower as a “mercy killer”

**Fig. 3:**
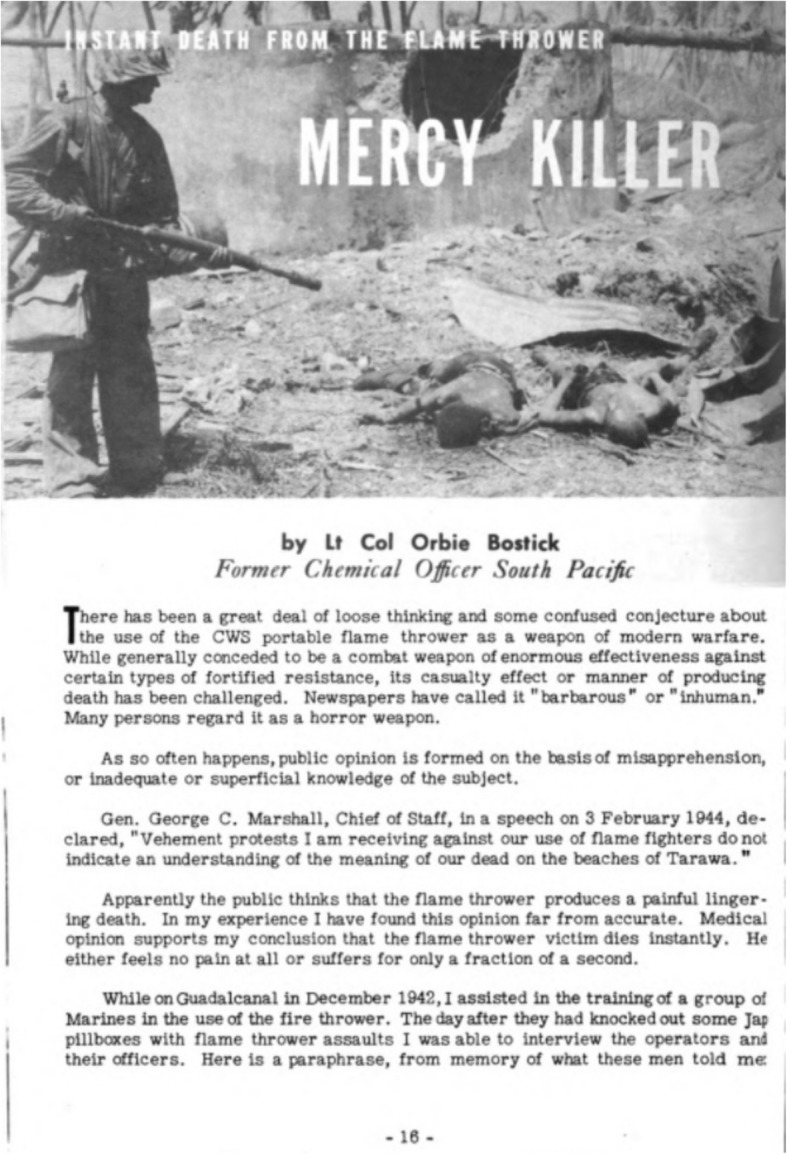
The cover page of the Feb-Mar 1944 *Chemical Warfare Bulletin* article written by Lt. Col. Orbie Bostick arguing that the flamethrower is an instant “mercy killer.” The Marine in the photograph is carrying an M1 flamethrower and is inspecting the remains of several Japanese soldiers following a flame attack

In 1944, the CWS began to run articles in the *Chemical Warfare Bulletin* proposing that flamethrowers caused instant death with far less pain and suffering than bullets, explosives, and other common battlefield weapons. Lt. Col. Orbie Bostick, a chemical officer in the South Pacific and author of a 1944 article entitled *Mercy Killer: Instant Death from the Flame Thrower* (Fig. 3), recounted an interview with Marine Corps flamethrower operators who had used the weapon to neutralize a Japanese pillbox during the campaign. Bostick wrote that the Marines had told him that “less than two min after the flame thrower bursts entered the gun ports we were examining the interior. We found five Jap bodies in the pillbox. Not one showed a spark of life. Those Japs were knocked out instantly, no question about it.” He then referenced his own personal experiences witnessing deaths from flamethrowers and noted that “I can vouch for the fact that the bodies of flamethrower victims were scarcely marked by flames; the skin was hardly touched.” Bostick suggested several mechanisms for what he calls the “instant-death theory” based on off-the-record discussions with personnel from the CWS Medical Division, the Surgeon General’s Office, and the Navy Department. He paraphrased their positions in his article and concluded that systemic shock was the cause of death: “The physiological effects of flame thrower attacks are complex. Principally, there is mass stimulation of the sensory nerve endings. This may also be described as a ‘profound insult’ to the nervous system. There is also intense shock to the respiratory center, paralyzing the muscles of respiration. It is believed the victim probably feels no pain.” He further concluded that based on these suppositions and experiences, the flamethrower is a “more merciful” weapon that many others, such as high explosives, since it leads to instantaneous death [6].

Not long after Lt. Col. Bostick published his article, the *Chemical Warfare Bulletin* ran another article, *CWS Studies Flame Deaths,* which continued to promote the idea that flamethrower deaths were instantaneous. As before, the author speculated as to the exact cause of death: “Profound shock to the nervous system, lack of oxygen in the blood (anoxemia), carbon monoxide poisoning, superheat generated at the flame front, third-degree burns and suffocation as the result of searing the air passages and lungs are the principle theories which have been advanced.” The article referenced a special report submitted by Captain J.F. Olds, a chemical officer during the New Georgia campaign who was charged with investigating whether the enemy died “a slow death or whether death was instantaneous.” Olds reported that enemy dead were found in some cases with severe burns and in other cases without signs of burning, that he had not seen enemy soldiers leave a fortification alive after a flamethrower attack and that “the enemy may or may not give a short cry when he encounters liquid flame [and] it is believed by all those who have operated flamethrowers or been at the action and who have examined the results that if such a cry did occur the enemy was dead by the time he finished the cry.” Ultimately, Olds concluded that “there is no lingering death when the flame hits the enemy” and that “we definitely know that death comes instantly [and] whether it be by high temperature or burning, the enemy dies a humane death [7].” Despite the absurdity of such a claim through a modern lens, there was little scientific evidence at the time to refute it.

### Studying the physiological and toxicological effects of flamethrowers

On March 22, 1944, the Technical Division of the CWS in Washington D.C. called a meeting to discuss “deficiencies” in the understanding of the casualty-producing effect of flamethrowers. It was noted that “little or nothing was known regarding the precise manner by which immediate disability or death is produced incident to flame thrower action,” and a recommendation was made that the Physiological Section of the Office of Scientific Research and Development (OSRD) Division 9 (Chemistry) “undertake an experimental investigation of the relative importance of casualty-producing attributes of flame thrower attack [8].” The purpose of doing so was to maximize the offensive use of flamethrowers in combat, as well as to develop effective defensive measures to protect soldiers against enemy flame attacks [9]. As a result, the CWS and NDRC organized and funded a series of investigations throughout 1944 involving multiple agencies, private companies, and universities. In general, the investigators began with the untested hypotheses that any single one or combination of the following mechanisms contributed to rapid fatality from a flamethrower attack: 1) the inhalation of carbon monoxide (CO), 2) hypoxia, 3) the inhalation of vaporized gasoline or intermediary combustion products, 4) the inhalation of superheated air, or 5) the burning of the surface of the body. Subsequent testing, therefore, was designed primarily to evaluate these factors [10].

**Fig. 4:**
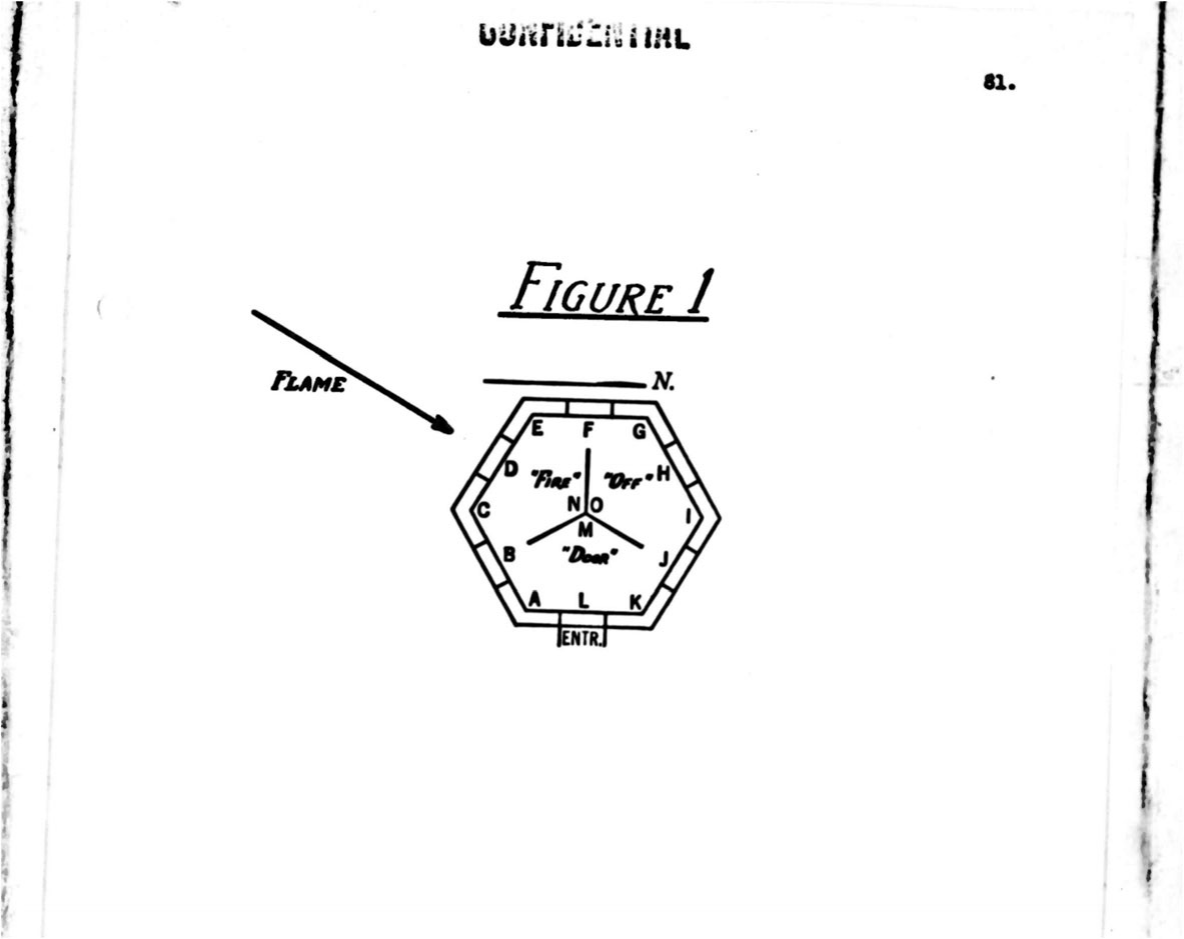
Diagram of the German-style bunkers constructed by Arthur D. Little, Inc. for testing designed to compare the effects of EWP and napalm-gas mixtures. Photo from Wheeler TL, and Bogrow A. Phosphorus-Phosphorus Sesquisulfide Eutectic as a Special Flame Thrower Fuel. OSRD 5523, OEMsr-242, Service Project CWS-10, Arthur D. Little Inc. August 3, 1945. PDF Retrieved from www.dtic.mil

Some of the earliest scientific data regarding the toxicological and physiological effects of flamethrowers came from testing intended to evaluate other aspects of portable flame warfare, such as new flamethrower equipment, fuels, and tactics. For example, Arthur D. Little, Inc. of Cambridge, Massachusetts, under Contract OEMsr-242, recorded the physiological effects of flame attacks against occupants within an enclosed structure while evaluating a new type of flamethrower fuel, phosphorus-phosphorus sesquisulfide, also known as EWP, in August 1944. The evaluators built a series of pillboxes modeled after German designs that included a reinforced concrete, earth-filled, hexagonal bunker with internal lengths of 6 ft on each side and a ceiling of 6.5 ft. The total inside volume measured 900 cubic feet, and the combined ventilation area included a 3.5 × 3.0-ft door and six 14.5 × 6.5-in. loopholes for a total of 14.5 square feet. A Y-shaped baffle of cinder blocks reached from the floor to the ceiling, with each baffle leg being 32 in. wide and 3 in. thick and radiating out from the center of the structure. This effectively divided the structure into three sections known as the “fire” (where the loopholes to be targeted for attack were located), “off,” and “door” compartments (Fig. 4). The M2–2 flamethrower was used to deliver a 1-gal flame attack from a fixed distance with either a 4% gasoline-napalm mixture or EWP. Small domestic swine with pneumographs, thermocouples, specialized discs that measured caloric bombardment, and electrocardiographs attached were placed into the pillboxes. Temperature and gas compositions were measured continuously within the pillbox throughout the experiment. In a second experiment, rabbits and pigs (one of each) were placed in six designated locations within the pillbox to determine which positions were most affected by 1-gal flame attacks from a fixed distance. The animals were removed from the pillboxes at the end of the experiment for autopsy and microscopic tissue evaluation. With regard to animal casualties, the investigators noted that thermal injury appeared to be the primary cause of death in the experimental animals, with respiratory conditions determined to be a contributing factor. Not surprisingly, burns were worst in the animals placed in the “fire” compartment, followed by the “off” and then “door” compartments, with animals in higher positions being more seriously injured than those in lower positions. Respiratory lesions, on the other hand, did not appear to be related to the animals’ position in the bunker and consisted mainly of atelectasis, pulmonary edema, and pulmonary hemorrhage [11, 12]. While these early reports demonstrated the flame’s ability to heat an enclosed space and affect all targets in a bunker, including those not directly in the flame’s path, further research later provided a more comprehensive understanding of flamethrower lethality.

On January 29–30, 1945, Division 9 of the NDRC organized and hosted the “Symposium on the Toxicological Aspects of the Flame Thrower” at Dumbarton Oaks in Washington, D.C. as a result of a request from the Medical Division of the CWS for assistance in evaluating the lethal effects of heat and other toxic factors associated with flame thrower attacks. The Symposium’s reports consisted of over a year’s worth of data collected by various research teams that, in summary, showed “the lethal effects of flame attack when used against enclosed spaces are not limited to burning, or even to circumambient heat, but include other factors the field importance of which is not yet fully determined [13].” One of the major reports on flamethrower casualties came from a Harvard University team led by Frederick C. Henriques, PhD and Alan Moritz, MD of Harvard University. A report of their study, “An experimental investigation of the physiological mechanisms concerned in the production of casualties by flame thrower attack” was compiled in August 1944 and presented at the symposium. The report detailed the effects of gasoline conflagration on several animal models. For their study, dogs and pigs were exposed to burning gas after being anesthetized and fastened to an iron frame 54 in. above the floor in the center of a conflagration room. From their experiments, they noted several key toxicological and physiological findings. The average temperatures from gasoline conflagration in ventilated and unventilated structures varied between 350°Cand 600 °C, though temperature peaks as high as 1000 °C were recorded. Direct exposure of the animal’s body to gas flames resulted in death from the effects of the heat within 60 s, though indirect exposure to the flames and the resulting increase in temperature had a variable effect and seemed related to the animal’s core temperature recorded by rectal thermometer. Animals that died within 30 min after exposure had systemic hyperthermia of 43 °C or higher, whereas those that survived had core temperatures measured at 42.5 °C or less. Additionally, the investigators found that all animals exposed to temperatures greater than 105 °C for more than 3 min, greater than 150 °C for more than 2 min, or greater than 400 °C for more than 30 s all sustained severe burns and became critical casualties. Physiological derangements noted in test animals included electrocardiographic changes such as tachycardia, inverted T-waves, a reduction in QRS voltage, and premature ventricular contractions that evolved into ventricular tachycardia and ventricular fibrillation. Blood pressure rose initially with exposure to heat, but a terminal drop usually occurred in conjunction with respiratory failure. Although potassium levels were elevated following liberation from the affected red blood cells and were suspected to be a cause of immediate death, the potassium levels never rose to a level that was considered likely to contribute to death in the animals. Despite this conclusion, the researchers found an overall poor correlation between cutaneous injury and physiological changes, with some of the most severe physiological derangements noted in animals with minor or no cutaneous injuries. Ultimately, the investigators concluded that rapid elevation in circulating blood temperature leading to the cessation of the cardiac function was likely the cause of immediate death following exposure to heat. Shock was also noted to be a major contributing factor in animals who did not die immediately. Interestingly, asphyxiation and CO poisoning were believed to be less likely than heat to cause immediate or rapid fatalities. The mean concentration of CO in the atmosphere of an unventilated room after the fires had burned out was 0.8%, and the mean oxygen concentration was 14.6%. One animal tested had a blood CO level of 30%. Thus, while CO exposure would likely have killed the animal if it had remained in the room for a long enough period, it was not thought to be a major contributor in these test subjects [14].

One potential criticism of studies such as those by Henriques and Moritz is that data obtained from animals may not extrapolate well to humans. Even pig skin, which is considered very similar to human skin from an anatomical perspective, lacks the human cooling mechanism of sweating. This characteristic could theoretically result in differences from the animal data, though the authors noted that a 70-kg male sweating maximally (approximately 1 l of sweat per hour or 1 mg of water per square centimeter) would require a caloric input of 0.6 cal/minute to evaporate this volume. Because the amount of caloric input to burn pig skin was between 0.8–45 cal/(g·min), depending on the degree of heat the animal was exposed to, the protection afforded to humans via perspiration was thought to be “trivial.” The pig data on caloric bombardment was therefore considered directly applicable to humans [15].

While Henriques and Moritz reported the effects of heat exposure on flamethrower casualties, other investigators presented data at the symposium on contributory factors such as asphyxia, CO poisoning, and hydrocarbon vapors. Contrary to some of the findings in the Contract OEMsr-242 study by Little, Inc., which downplayed the respiratory effects of flame attacks, these studies noted several major findings independent of the thermal injuries. Lieutenant (JG) J. Seronde of the Naval Reserve Medical Corps reported on several corpses examined after flamethrower attacks during the Battle of Saipan in which respiratory injury appeared to play a significant role. Specifically, he reported that four civilian women who had been found hiding in caves with Japanese soldiers were subjected to flamethrower attacks and had no external burns but appeared to have died as a result of respiratory failure, with postmortem exams showing evidence of inhalational injury caused by irritant substances derived from flamethrower fuels [16]. This seemed to complement findings of ventricular fibrillation in test animals that appeared secondary to hydrocarbon vapors reported by 1st Lieutenant M.B. Chenoweth from the Medical Division of the CWS [17]. Cardiac toxicity from hydrocarbon exposure has since become a well-documented consequence of recreational use of hydrocarbon substances such as “huffing” which involves breathing a rag soaked with a hydrocarbon inhalant such as kerosene, gas or naphtha [18]. Further, some investigators reported a rapid and sudden increase in lethal CO levels detected within the bunkers, as well as dangerously low oxygen content. These reports noted that when carboxyhemoglobin levels in the blood reached 70%, the result was unconsciousness and usually death and that CO could reach lethal levels in poorly ventilated structures within 2 min. Furthermore, as little as 0.1% CO in breathable air was sufficient to maintain lethal blood levels of CO, and such levels could be remain for 7–10 min after a flame attack before beginning to decrease. At the same time, oxygen was completely depleted for up to 15 s, a more than adequate time for instantaneous unconsciousness to result [19–21]. These studies suggested that respiratory factors were a significant contributing cause of death for those in an enclosed space under flame attack. The findings are generally consistent with what is reported in contemporary medical literature, i.e., that carboxyhemoglobin levels higher than 60% are generally fatal [22]. Furthermore, these studies brought attention to the effects of inhaling superheated air and smoke. Current data have shown that temperatures within an enclosed burning structure can reach 540–1150 °C. Breathing air at these temperatures results in burns of the oropharynx, leading to progressive upper airway edema and, ultimately, asphyxia. This is worsened by the water-soluble toxins present in smoke that precipitate in the trachea and further degrade pulmonary function by causing chemical tracheitis and/or pneumonitis [23, 24]. These inhalation injuries and the rapid development of airway edema may explain the brief cries of flamethrower victims noted in the *Chemical Warfare Bulletin* articles. Although airway edema and tracheitis would have significantly muffled any sounds made by the casualties, this would not have resulted in the instant, painless death proposed by some researchers.

**Fig. 5:**
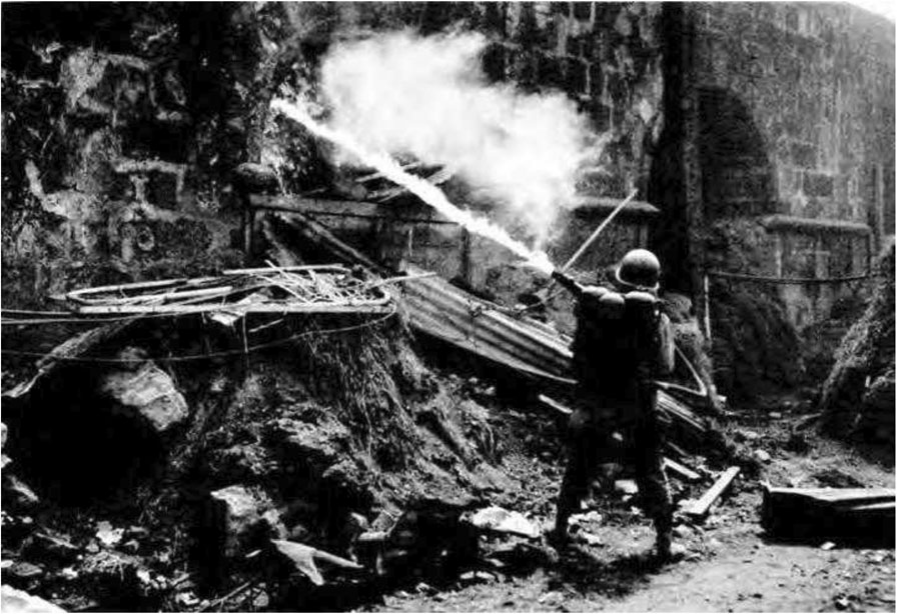
A U.S. Army flamethrower operator fires his M2–2 into a wall opening in Manila, Luzon, February 1945. Note the thin fire stream that indicates that napalm was added to the mixture. Photo from Brophy, Miles and Cochrane, The Chemical Warfare Service: From Laboratory to Field, Department of the Army, 1959

The use of fuel thickeners, namely, napalm, in later models was also found to have increased the lethality of flamethrowers (Fig. 5). While the M1 and M1A1 models could not effectively use fuel-thickening agents because the discharge piping and barrel in the wand (the gun) were too small, the M2–2 was designed to accommodate thickeners that nearly tripled the weapon’s effective range [4]. From a toxicological perspective, however, the addition of napalm-thickened fuel results in the creation of localized areas of elevated carbon dioxide levels in enclosed spaces. Carbon dioxide levels can reach as high as 20% in the presence of burning napalm [25], with levels > 10% being known to cause rapid deoxygenation, loss of consciousness, convulsions, and death [26]. Furthermore, studies presented at the symposium evaluating the effects of thickened fuels showed that, even from short ranges, when compared to a liquid fuel, a larger amount of thickened fuel enters a fortified position [27]. These studies were important in shaping our modern understanding of asphyxiation, inhalational injury, and CO poisoning.

Ultimately, the symposium succeeded in identifying factors that were, either alone or in some combination, both known and probable contributors to death following flame attack. Known contributors included 1) heat acting rapidly via incineration or the induction of hyperthermia or slowly through shock, various toxicities, or infection; 2) asphyxiation or carbon monoxide poisoning; and 3) pulmonary edema. Probable contributors included irritant effects from smoke and ventricular fibrillation from multiple potential sources (i.e., hydrocarbons, asphyxiation, etc.) [28]. It also became clear that death, while potentially rapid, was unlikely to be instant or painless in many cases, making it difficult to perpetuate the concept of humane deaths from flame attacks.

### Revised perceptions of flamethrowers

With the flamethrower reaching the pinnacle of its service life in WWII, scientific evaluation and first-person accounts of its use have allowed for a better understanding of the direct and indirect effects of flame attacks. The physiological and toxicological effects of flame attacks on human targets are devastating and terrible. In retrospect, it is fair to say that Lt. Col. Bostick’s “instant death theory,” while perhaps accurate under specific circumstances, does not have widespread applicability. The International Committee of the Red Cross—Conference of Government Experts on the Use of Certain Conventional Weapons demonstrated in 1975 the controversy surrounding the use of flame warfare. Experts generally agreed, for example, that mortality among burn victims was variable and related to the extent and degree of burns, the age and physical condition of the victim, and whether comprehensive and rapid medical care was available. Many also believed that burn wounds were more complicated and painful than mechanical wounds considering the repeated medical and surgical interventions often required to treat severe burns. Likewise, most agreed that asphyxiation and CO poisoning were possible when burning occurred in enclosed spaces and that both were more likely with napalm considering the large amount of oxygen consumed and CO and carbon dioxide produced by burning napalm. Several examples of survivors of napalm accidents were discussed, including victims who were “right in the fireball”, as were studies citing battlefield mortality rates as a result of burns of all types being approximately 35%. In the end, a number of experts at the conference concluded that incendiary weapons, including flamethrowers, cause extreme and unnecessary suffering when compared to other weapons [29]. Chris McNab, author of “The Flamethrower”, summarizes the contemporary view of flamethrowers best in a most condemnatory fashion: “All weapons are, by their very nature, ghastly in purpose but there is something uniquely awful and inhumane about the flamethrower. Fire is popularly and quite logically held as one of the worst ways to die. It is not quick, the victim taking long seconds or even minutes to succumb, as the flesh, nerve, muscle and eventually organs are charred to destruction. That intelligent human beings have turned their minds to developing instruments specifically to achieve this goal represents another tragic failure in society [30]. These evolving views on flame warfare were ultimately factors considered in the U.S. military’s decision to voluntarily withdraw flamethrowers from active service in 1978. Nevertheless, a number of other nations still stock flamethrowers in their military inventories, and there is no international law specifically banning their use in war [31].

## Conclusions

The suggestion that flamethrowers were once viewed as instantaneous “mercy killers” is difficult for many to comprehend today. The gross misunderstandings and mischaracterizations of flamethrowers were likely the result of a number of factors, with one possibly being an effort by the military to assuage the horror young soldiers must have felt upon hearing the screams and seeing the charred remains of enemy soldiers following flame attacks. However, another reason was quite clear from the lack of scientific data detailing exactly how flamethrower casualties were affected by the weapon. The flamethrower’s history illustrates the importance of understanding the scientific aspects of a weapon’s killing mechanisms and lethality not only to maximize its use on the battlefield but also to apply what is learned to military medicine in a broader sense so that effective countermeasures and treatments for soldiers can be developed and utilized.

## Data Availability

Not applicable.

## Abbreviations

CO: Carbon monoxide
CWS: Chemical Warfare Service
NDRC: National Defense Research Committee
WWI: World War I
WWII: World War II
